# KEEPING THINGS, BUT ONLY FOR A WHILE

**DOI:** 10.1093/geroni/igz038.1323

**Published:** 2019-11-08

**Authors:** David J Ekerdt

**Affiliations:** University of Kansas, Lawrence, Kansas, United States

## Abstract

The life course is accomplished by material culture held as a convoy of possessions, but also sustained by public affordances and amenities that include the artifacts and artworks to be found in museums. In both places—household and museum—objects come and go, but there is mainly keeping. The difference lies in the capacity to keep things indefinitely: it is virtue for museums but a predicament for households of aging adults. Museums model ideals of permanence and responsibility toward things, ideals that, in the long run, households can only faintly attain. For older adults and for gerontologists, preservation is the wrong lesson to take away from the galleries. Rather, what we can learn there is how single, selected things can show, in a thoughtful way, an entire world of ideas and universe of meaning. No need to keep it all—and forever—but we can honor things while we can. ​

